# Monthly Summary

**Published:** 1878-04

**Authors:** 


### MONTHLY SUMMARY.

---

*Is the Human Eye Changing its Form, and becoming Near Sighted, under the Influence of Modern Education?*—Dr. E. G. LORING recently read an interesting paper on this subject before the New York County Medical Society (*New York Med. Jour.*, Dec. 1877.) He said that hereditary influence was an important element in the production of myopia, and, although statistics did not strongly indorse that view, he still held that legendary information should receive much credence. In regard to the influence of modern education, it was found that a larger proportion of those living in cities were near-sighted than those in country districts; and, moreover, in those cities where intellectual pursuits were greatest, the largest number of myopes were found. In savage nations near-sightedness was very infrequent, and it would seem, in some respects, that it was a result of education. While the intellectual classes in Germany showed a large proportion of myopia, it was not so found in those artisans who used their eyes on fine objects, as watchmakers and wood-engravers. In England, where there has always been great intellectual activity, by no means as large a ratio of near-sightedness had been detected as in Germany, and it became necessary to seek for other factors to explain the prevalence of myopia. Impaired nourishment, imperfect ventilation, together with a sedentary life, had a marked tendency in producing laxity of the tissues in general, including of necessity the coats of the eyeball; and, with the tension which resulted from close application of the sight, there was a great probability of lengthening of the eye, or myopia, resulting. In New York the German children were found more often near-sighted than those of other nationalities. Dr. Loring said that undoubtedly myopia was hereditary, but that in all probability it could under no circumstances be developed; but he did not believe that of necessity it must increase in a nation engaged in literary pursuits. In the United States the normal eye predominated, and he thought it was due to the fact that the young were more in the habit of indulging in out-door sports than in Germany. The same was true of England. From a careful analysis of the myopic cases, it was found that between the ages of ten and fifteen the majority developed; or, in other words, at that time the tissues of the globe were more readily affected by strain of the muscles of the eye. It could be easily understood, under such an hypothesis, why the industrial classes were so little liable to near-sightedness, for they seldom reached the practice of the more intricate branches of their trade before their eighteenth year. In conclusion, Dr. Loring was of the opinion that under proper precautions, the normal eye could be continued indefinitely. If children were not allowed to apply themselves too closely to their studies between their eighth and sixteenth years, and were, moreover, allowed the proper amount of outdoor exercise, not much danger need be dreaded. It was important also to have the schools perfectly ventilated, and other hygienic conditions made as perfect as possible.—*Medical News and Library.*

---

*Simple Mode of Relief for Foreign Bodies in the Throat.*—A British naval surgeon, Dr. Beveridge, states that for foreign bodies in the throat, such as a piece of meat, etc., a simple mode of relief is to blow forcibly into the ear. This excites powerful reflex action, during which the foreign body is expelled from the trachea. The plan is so easy of execution that, if there is anything in it, it ought to be generally known and applied —*Canada Medical Record.*

---

*Danger of Ether.*—The following painful occurrence is related by the Paris correspondent of the New York *Times.* Whatever may be the dangers attending the application of chloroform, it has at least the advantage of not being inflammable.

A young lady of eighteen, remarkably beautiful, belonging to a family of rich merchants of Lyons, had to undergo a surgical operation. The surgeon said that it was necessary to give her ether. The sack was prepared, and the young lady had been inhaling it for a moment, when a light was brought near the patient. In an instant the ether was ignited, and the sack exploded. The doctor was himself seriously burned, but the young lady was in a lamentable condition. Her nose was taken off completely, and one side of the upper jaw was laid bare. It is needless to say that she is horribly disfigured for life. No one could describe the despair of the family, and perhaps it would have been better had the poor girl died from the effects of this dreadful wound. It is rumored that the doctor has committed suicide.—*Druggists Circular.*

*Injury from Disturbed Sleep.*—"It is diseased imagination which fills our mad houses. * * It is *expectation* which deranges the normal condition of the brain. * * The difference in annoyance [as of disturbed rest] is immense as between the well man and the sick; moreover, there is also a difference in this connection, not only between those who are ill and those who are well, but a difference, and a natural one, between men and women. * * To invalids the afternoon or early evening sleep may be as important as the night sleep, and in the case of infants particularly so. * * To many men the hour or two of early rest on Sunday morning is a necessity, not a luxury. The pressure of modern social life necessarily produces, especially among professional men, a degree of brain-tire—of loss of power to use the brain, of which the results are terribly alike, beginning with insomnia, irritability, nervous excitement, cerebral derangement, and running the gamut of mischief down to paralysis and death. * * A man whose brain has been sorely worked during the week, and whose brain-tire is habitually lessened by one or more extra hours of sleep on a Sunday, is pushed well on his way to disease by having that natural medicine withdrawn."—*S. Weir Mitchell, on the ringing of St. Mark's, Philadelphia, Chimes, and the disturbance to sleep of the neighborhood.—Jour. of Nervous and Mental Diseases.*

---

*A New Counter-Irritant.*—It is related by Dr. Coutivier, in *L' Union Medicale*, that the extract of pimento is a most admirable revulsive in a large number of cases, and may usefully take the place of mustard and flies. It is proposed by M. Lardy. The writer says it acts with great rapidity, ten to thirty minutes, according to the point of application and the delicacy of the skin. Its action is manifested at first by a sensation of heat, a slight smarting and redness. These go on increasing for about three hours, then they remain stationary, and the revulsive action is so continued as long as may be desired. Nevertheless, after twenty or twenty-four hours in the adult, eight to ten in children, it is better to remove the plaster, and put another alongside of it if it be desirable to continue the revulsion. The heat and tingling produced are painless and free from itching. * * * * The extract of pimento has a beautiful red color identical with that of the dried fruit. Suitably incorporated in a plastic mass, and spread upon squares of paper, its application is very easy. It is unnecessary to warm it, for it adheres sufficiently to the skin; but it is well, on parts subject to movement, to fix it with a bandage just as a blister. Moreover, its action may be augmented or moderated according to the pressure. On removal, the heat and tingling may be immediately arrested by the application of a little starch.—*Druggists Circular.*

*Death from a Carious Tooth.*—A singular case of death lately took place at the Lariboissiére Hospital, under the following circumstances: The deceased, a boy aged seven, was admitted into the hospital complaining of pain in the head accompanied with a tendency to stupor. His eyes were somewhat prominent, but nothing else remarkable was noticed in the patient. About a month after admission coma set in, and this was accompanied with high temperature, which carried him off. A small, hard body was felt over the right eye, which was supposed to have been the cause of death. At the post-mortem examination the real cause was traced to a carious tooth, one of the inferior molars, which caused an abscess in the jaw. The inflammatory process extended along the dental nerve, entered the skull through the orbit, involving the dura mater and the brain. The latter contained two or three abscesses, and the bones of the skull were necrosed to a considerable extent. An abscess was also found in the heart. This case would show how important it is to attend early to carious teeth, and to have them removed as soon as inflammatory symptoms become evident, and not to tamper with them by "stopping" or other useless means frequently resorted to by dentists, to the detriment of the patient's pockets, if not of their health or life.—*British Medical Journal.*

---

*A Simple Telephone.*—Professor Barrett, in a recent lecture on the telephone, gave a recipe for making a cheap one. Take a wooden tooth-powder box and make a hole about the size of a half crown in the lid and the bottom. Take a disk of tinned iron, such as can be had from a preserved meat tin, and place it on the outside of the bottom of the box, and fix the cover on the other side of it. Then take a small bar magnet, place on one end a small cotton or silk reel, and round the reel wind some iron wire, leaving the ends loose. Fix one end of the magnet near, as near as possible without touching, to the disc, and then one part of the telephone is complete. A similar arrangement is needed for the other end. The two are connected by the wire, and with this Professor Barrett says that he has been able to converse at a distance of about 100 yards.—*Druggists Circular.*

---

*Calculus in the Sublingual Duct.*—Dr. F. D. Lamb, of Great Bend Village, Pa., reports a case of the above occurring in a young man, aged 26 years. It appeared as a suppurating swelling under the tongue, which discharged pus and disclosed a white and hard substance through the opening. The latter was enlarged, and the calculus, one-fourth of an inch in length, one-tenth of an inch in diameter, and weighing six grains, was removed.—*Med. Record.*

*Results of Intermarriage.*—Mr. George Darwin, after searching investigation, concludes that "the widely different habits of life of men and women in civilized nations, especially among the upper classes, tend to counterbalance any evil from marriage between healthy closely related persons." Mr. Darwin's views are in a measure sustained by Dr. Vorni's inquiry into the commune of Batz. Batz is a rocky, secluded, ocean-washed peninsula of the Loire Inferieure, France, containing over three thousand people of simple habits, who don't drink, and commit no crime. For generations they have intermarried, but no cases have occured of deaf-mutism, albinoism, blindness, or malformation, and the number of children born is above the average.—*Med. and Surg. Reporter.*

---

*American Dentists in Europe.*—At the meeting of the New York Dental Society, Dr. Straus, of Paris, made the following remarks:

"I have a few remarks to make in regard to the dentists on the Continent. In the first place, in regard to the fees. I have noticed that among the better class of dentists there, speaking principally of Americans, as a matter of course, and among the better class of patients which I have had occasion to notice in St. Petersburg, Berlin, Vienna, Paris and London, that the fees are uniform with the fees of first-class dentists here. The prices vary from five dollars to fifty dollars for filling teeth, and not less, but rather more. Artificial work pays better in general. In France an American will not put in a set of teeth for less than eight hundred francs, which, I believe, is about one hundred and fifty dollars—a little over. The art of dentistry is, in general, among the better class of people in Europe, as much esteemed and looked for as here in America. Your next meeting, I believe, is in October. I could then with the greatest pleasure give you a fuller idea of my experience in dentistry of the last ten years in St. Petersburg and in other cities which I have named. * * * The native dentist has not the standing that the American dentist has in Europe, in England or in France. There are some exceptions, such as the dentist to the Prince of Wales. Those dentists are reputed as a matter of course, and from those persons they get first-class prices; but, as a general thing, the prices are much better in Europe all over—it must be borne in mind I am speaking of the aristocrats in Europe—they pay better than in America."

---

*New Anæsthetic.*—Recent tests of the new anæsthetic, carbon tetrachloride, prove it to be more energetic than any previous agent used for the purpose, and perfectly regular in its action.

*The Purity of Chloral.*—At a recent meeting of the Pharmaceutical Society of this city, Prof. Maisch said that, from some experiments, he was convinced that the shape of the crystals was no criterion of its purity; that pure chloral hydrate had a slight acid reaction, and that the density of the white vapors produced with a glass rod moistened with ammonia was largely influenced by the temperature. The practice of giving a little information about physical properties, for the purpose of influencing trade, was carried on in Europe as well as here; he did not believe that absolutely pure chloral hydrate had as yet been put into the market, and he was strengthened in this belief by the transactions of the Berlin Apothecaries' Society, where this question was incidentally ventilated. Of late chloral chloroform, that is, chloroform made by the decomposition of chloral, had been bruited in Germany as the only article worthy of confidence for its purity, but the researches that have been instituted by Schacht and Bilz, upon this claimed superiority of chloral chloroform, had shown it to be entirely erroneous, as the chloral chloroform when treated with sulphuric acid became discolored very speedily, like the chloral from which it had been prepared, which is not the case with absolutely pure chloral, or with the chloroformum purificatum of the Pharmacopœia.—*Med. and Surg. Reporter.*

---

*Death from Chewing Tobacco.*—A boy was recently admitted into the Melbourne Hospital suffering from the effects of chewing tobacco. He was in a very weak state, and complained of severe headache and dysentery. He became gradually unconscious and partially paralyzed, and died on the following day. The cause of death was narcotic poisoning.—*Druggists Circular.*

---

*Transplantation of Teeth.*—On the 14th of May last I inserted a right superior central incisor, which had been extracted for more than a year, into an alveolar socket from which I had just extracted a like tooth for another patient. The tooth still remains firm in the socket, and the patient tells me that he does not know any difference between that and his other teeth.—E. H. Locke, in *Dental Cosmos.*

---

*Eucalyptus as a Local Anæsthetic.*—Dr. Horton (Ohio State Dental Society) speaks of the extract of eucalyptus as producing good results as a pain obtunder in sensitive dentine. A drop on a pledget of cotton is used. He thinks it the best of the local applications.

*Cyanide of Zinc in Facial Neuralgia.*—Dr. Luton, of Rheims, states that he has obtained excellent results from the cyanide of zinc in rheumatic facial neuralgia simulating cerebral rheumatism. He relates two cases in which, with intense facial neuralgia, there was continued and ardent fever, cephalalgia and tenderness, on pressure at the points where the nerves emerged. The symptoms rapidly abated under the use of the following mixture:—Cyanide of zinc one-fifth of a part, distilled cherry-laurel water twenty-five parts, and tragacanth mucilage mixture 100 parts. A tablespoonful, from hour to hour.—*Journal of Mat. Medica.*

---

*Mixed Narcosis.*—During the past two or three weeks, says the *Lancet*, December 1, a novel mode of producing anæsthesia, called mixed narcosis (*gemischte narkose*) has been employed by Thiersch, of Leipzig, whereby insensibility to pain may be procured without the total abolition of consciousness. The credit of the discovery is ascribed to Professor Nussbaum, of Munich. Although suitable for all kinds of operations, it is especially serviceable for operations about the mouth and jaws, in which blood is apt to flow into the trachea, or down the œsophagus into the stomach, and subsequently to cause vomiting. In some cases of removal of the upper jaw lately performed by Thiersch, the patient allowed the blood to accumulate for a while at the back of the pharynx, and then spat it completely out when asked to do so; and we are informed that in one instance the patient watched with evident interest the motion of the saw that was dividing his upper jawbone.

A subcutaneous injection of morphia, from a quarter to half a grain, is given as soon as the patient is placed upon the operating table, and immediately afterward the administration of chloroform is commenced. After inhalation for about five minutes the operation may usually be begun, but the chloroform must be renewed at intervals. The patients lose all sensibility to pain, but evidently retain a considerable degree of consciousness and control of voluntary movements. Within the last month mixed narcosis has been employed five times successfully, as far as the annihilation of pain is concerned, and without any bad effects.

